# Proteomic Signatures as Biomarkers of Atherosclerosis Burden

**DOI:** 10.21203/rs.3.rs-6837440/v1

**Published:** 2025-06-10

**Authors:** Lanyue Zhang, Murad Omarov, LingLing Xu, Barnali Das, Hong Luo, Stefanie M. Hauck, Agnese Petrera, Zhi Yu, Sascha N. Goonewardena, Eleftheria Zeggini, Annette Peters, Martin Dichgans, Venkatesh L. Murthy, Barbara Thorand, Marios K. Georgakis

**Affiliations:** 1 Institute for Stroke and Dementia Research (ISD), LMU University Hospital, LMU Munich, Munich, Germany; 2 Urological Diseases Research Center, Boston Children’s Hospital, Boston, MA, USA; 3 Institute of Epidemiology, Helmholtz Zentrum München, German Research Centre for Environmental Health (GmbH), Neuherberg, Germany; 4 Metabolomics and Proteomics Core, Helmholtz Zentrum München, German Research Center for Environmental Health (GmbH), Neuherberg, Germany; 5 Clinical and Translational Epidemiology Unit, Department of Medicine, Massachusetts General Hospital, Boston, MA, USA; 6 Broad Institute of MIT and Harvard, Cambridge, MA, USA; 7 Division of Cardiovascular Medicine, Department of Internal Medicine, University of Michigan, Ann Arbor, Michigan, USA; 8 Division of Cardiovascular Medicine, VA Ann Arbor Health System, Ann Arbor, Michigan, USA; 9 Institute of Translational Genomics, Helmholtz Zentrum München, German Research Center for Environmental Health, Neuherberg, Germany; 10 Technical University of Munich (TUM), TUM University Hospital, TUM School of Medicine and Health, Munich, Germany; 11 Institute for Medical Information Processing, Biometry, and Epidemiology, Faculty of Medicine, Ludwig-Maximilians-Universität, Munich, Germany; 12 Munich Cluster for Systems Neurology (SyNergy), Munich, Germany; 13 German Center for Diabetes Research (DZD), Partner München-Neuherberg, Neuherberg, Germany; 14 German Center for Neurodegenerative Diseases (DZNE), Munich, Germany; 15 German Centre for Cardiovascular Research (DZHK), Munich, Germany; 16 Program in Medical and Population Genetics and Cardiovascular Disease Initiative, Broad Institute of MIT and Harvard, Cambridge, MA, USA

**Keywords:** proteomics, atherosclerosis, machine learning, risk prediction, cardiovascular disease

## Abstract

Atherosclerosis progresses silently over decades before manifesting clinically as myocardial infarction or stroke. Currently, no circulating biomarker reliably quantifies the burden of atherosclerosis beyond imaging techniques. Here, we sought to define plasma proteomic signatures that reflect the systemic burden of atherosclerosis. Using CatBoost machine learning applied to plasma proteomes (Olink Explore 3072; 2,920 proteins) from 44,788 UK Biobank participants, we derived four proteomic signatures which robustly discriminated individuals with known atherosclerotic disease from propensity score-matched controls (ROC-AUC up to 0.92, 95% CI: 0.90–0.94 in the test set). Each signature was based on distinct protein sets: the whole proteome (WholeProteome; n = 2920), proteins associated with genetic predisposition to atherosclerosis (Genetic; n = 402), those implicated in atherogenesis (Mechanistic; n = 680), and proteins enriched in arterial tissue (Arterial; n = 248). Among 41,200 individuals without atherosclerosis at baseline, all four signatures were strongly associated with future major adverse cardiovascular events over a median follow-up of 13.7 years (HR per SD increase in WholeProteome signature: 1.70, 95% CI: 1.64–1.77), providing significant improvements in risk discrimination (ΔC-index: +0.036; p <0.0001) and reclassification (Net Reclassification Index: 0.085–0.135 at a 10% risk threshold) beyond SCORE2. Signature levels increased with the number of clinically affected vascular beds, correlated with carotid ultrasound–measured plaque burden, and predicted future myocardial infarction and stroke in the external KORA S4 (n=1,361) and KORA-Age1 (n=796) cohorts with a median follow-up period of 15.1 and 6.8 years, respectively. Longitudinal analyses across three serial assessments showed that all signatures followed distinct trajectories, with significantly steeper annual increases among individuals with a higher burden of vascular risk factors. These findings demonstrate that proteomic signatures effectively capture atherosclerotic burden and improve cardiovascular risk prediction in asymptomatic individuals. Plasma proteomics may serve as a scalable and accessible alternative to imaging for identifying subclinical atherosclerosis, thereby supporting prevention strategies for cardiovascular disease.

Cardiovascular disease remains the leading global cause of death and disability,^1,2^ driven primarily by atherosclerosis,^3^ a progressive, lipid-driven inflammatory process that silently accumulates over decades before culminating in clinical events such as myocardial infarction and stroke.^4,5^ Despite advances in prevention and treatment, many individuals with a high atherosclerosis burden remain undiagnosed until the occurrence of a major cardiovascular event, underscoring a critical gap in early detection and prevention.^6^ The continued rise in the incidence of cardiovascular events^7,8^ further emphasizes the need to refine current paradigms of risk assessment.

Current prevention strategies rely on population-level algorithms, such as SCORE2 and the pooled cohort equations, which estimate cardiovascular risk based on demographic and clinical variables including age, sex, blood pressure and cholesterol levels.^9–11^ While widely adopted, these models do not directly quantify atherosclerotic burden and offer limited resolution in individual risk assessment. Direct detection of subclinical atherosclerosis remains dependent on imaging modalities including angiography, CT, MRI, PET, or ultrasound.^12–14^ While informative, these techniques are constrained by procedural risks, radiation exposure, availability, and the need for specialized personnel.^15^ Circulating biomarkers could overcome these limitations by offering scalable tools for identifying individuals with atherosclerosis, improving cardiovascular risk stratification, and facilitating longitudinal monitoring. However, existing circulating biomarkers, such as C-reactive protein (CRP)^16^ or cardiac troponins^17^, primarily reflect systemic inflammation or myocardial injury and fall short of directly assessing atherosclerotic plaque burden or progression.

Circulating proteins may serve as real-time indicators of pathophysiological processes.^18^ Recent advances in proteomic technologies enable the simultaneous quantification of thousands of proteins,^19,20^ providing a window into dynamic, tissue-specific pathophysiological processes. Integrating these data through machine learning has uncovered proteomic signatures predictive of early stages of neurodegenerative disease,^21–25^ cancer,^26–28^ diabetes,^29–31^ autoimmune disease^32,33^, and mortality risk through proteomic aging clocks^34^. While previous studies have shown potential for plasma proteomics in improving prediction of specific cardiovascular otucomes^35–41^, the capacity of plasma proteomics to systematically capture the burden and trajectory of atherosclerosis has not been fully elucidated, limiting its utility for assessing disease stage and extent.

Here, we leveraged plasma proteomics from the UK Biobank (UKB) and two independent cohorts to develop and validate four biologically informed proteomic signatures of atherosclerotic burden (AtheroBurden). Using machine learning, we constructed four signatures based on data from 1,666 cases with established atherosclerotic disease and 1,666 age- and sex-matched controls: WholeProteome (derived from the entire proteome), Genetic (genetically anchored proteins identified via Mendelian randomization), Mechanistic (proteins implicated in atheryogenesis), and Arterial (artery-enriched proteins). We evaluated the ability of these signatures to predict incident cardiovascular events in 41,200 disease-free UKB participants (median follow-up 13.7 years), and further validated externally in Cooperative Health Research in the Region of Augsburg (KORA) S4 (n=1,361, median follow-up 15.1 years) and KORA-Age1 (n=796, median follow-up 6.8 years). Subsequently, we analyzed associations between the signatures and carotid plaque burden measured by imaging. Finally, we assessed the longitudinal trajectories of these four signatures across three serial time points spanning a median of 12.5 years, and investigated how signature trajectories are influenced by baseline cardiovascular risk factors and the occurrence of future cardiovascular events.

## Results

### Summary of the study design

**Extended Figure 1.**
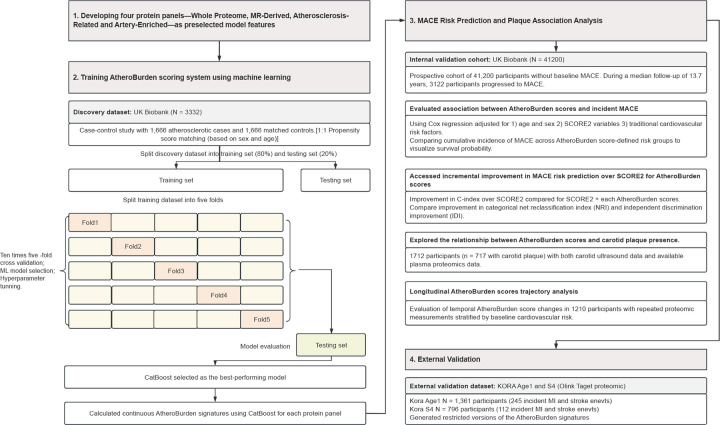
Detailed study workflow and analytical framework. This figure expands on the main workflow, presenting detailed steps of the study, including dataset characteristics, machine learning processes, and specific evaluation criteria for the AtheroBurden scoring system. Abbreviations: MR, Mendelian randomization; MACE, major adverse cardiovascular events; ML, machine learning; SCORE2, Systematic COronary Risk Evaluation 2; KORA, Cooperative Health Research in the Region of Augsburg.

**Extended Figure 2.**
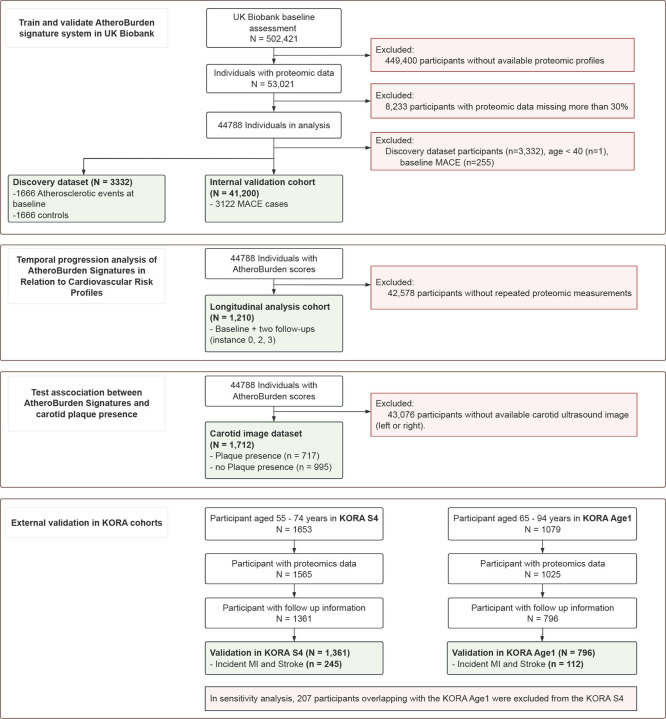
Flow chart of participant exclusions. Abbreviations: MACE, major adverse cardiovascular events; KORA, Cooperative Health Research in the Region of Augsburg; MI, myocardial infarction.

**Extended Figure 3.**
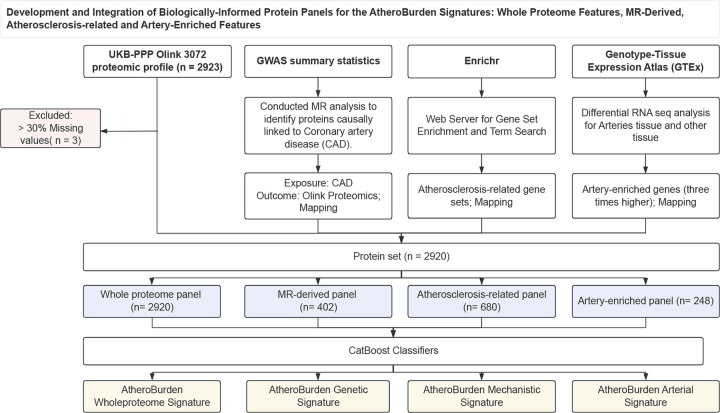
Systematic development and implementation of biologically informed proteomic panels for AtheroBurden score construction: (1) the entire plasma proteome, (2) proteins causally linked to atherosclerotic disease through Mendelian randomization approaches, (3) proteins with established roles in atherosclerosis pathogenesis validated through pathway enrichment analysis, and (4) arterial tissue-enriched proteins identified through tissue-specific expression analysis. Abbreviations: GWAS, genome-wide association study; CAD, coronary artery disease; MR, Mendelian randomization; UKB-PPP, UK Biobank Pharma Proteomics Project; GTEx, Genotype-Tissue Expression Atlas.

**Figure 1.**
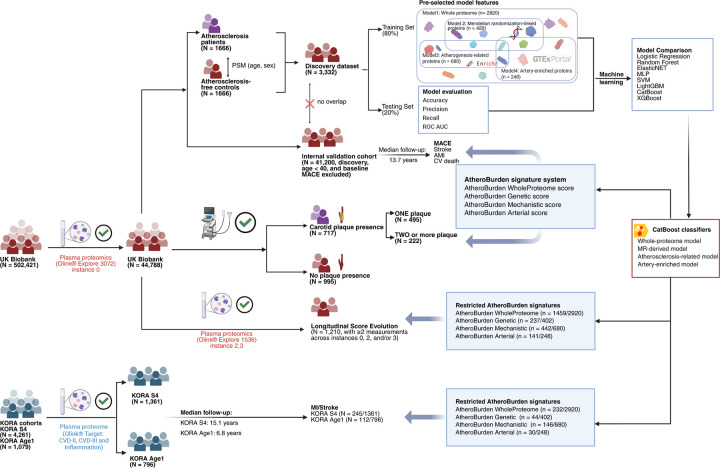
Overview of the study design and analytical approaches. The diagram illustrates the methodological approach implemented for protein-based atherosclerosis burden quantification. Machine learning models were trained in a UK Biobank discovery dataset (n=3,332; 1,666 atherosclerosis cases and 1,666 age- and sex-matched controls) using four biologically-informed protein panels. The resulting AtheroBurden signatures were validated in a disease-free UK Biobank cohort (n=41,200; median follow-up 13.7 years), assessed for association with carotid plaque burden (n=1,712), evaluated longitudinally (n=1,210), and externally validated in the KORA S4 (n=1,361) and Age1 (n=796) cohorts. Abbreviations: MACE, major adverse cardiovascular events; AMI, acute myocardial infarction; CV death, cardiovascular death; MI, myocardial infarction; ROC AUC, receiver operating characteristic area under curve; GTEx, genotype-tissue expression; MR, Mendelian randomization; MLP, multilayer perceptron; ElasticNET, elastic net regression; XGBoost, eXtreme Gradient Boosting; LightGBM, light Gradient Boosting Machine; CatBoost, categorical boosting; SVM, support vector machine; KORA, Cooperative Health Research in the Region of Augsburg.

The study design is summarized in Figure 1. A detailed study workflow, including data processing, ML model development, and validation steps, is provided in Extended Figure 1. Of the 502,421 participants enrolled in the UKB, a total of 44,788 participants (54% female, median age 58 years [interquartile range, IQR: 39–71 years]) met our inclusion criteria, after excluding participants with >30% missing proteomic data (Extended Figure 2). To develop proteomic signatures of atherosclerosis (AtheroBurden signatures), we leveraged four sets of proteins (Extended Figure 3) and trained ML models to discriminate the 1,666 cases with established atherosclerotic disease from 1,666 age- and sex-matched controls (discovery dataset). The developed AtheroBurden signatures were subsequently tested for associations with incident major adverse cardiovascular events (MACE, defined as a composite of myocardial infarction, stroke, or cardiovascular death) over a median follow-up of 13.7 years (n=41,200), followed by external validation in KORA S4 (n=1,361, median follow-up 15.1 years) and KORA-Age1 cohorts (n=796, median follow-up 6.8 years). Baseline characteristics of participants in the development cohort (UKB) and both validation cohorts (KORA S4 and KORA-Age1) are presented in Table 1. We further explored associations of the derived signatures with imaging evidence of atherosclerosis on carotid ultrasound (n=1,712), as well as serial changes across three timepoints and longitudinal progression patterns stratified by both baseline SCORE2 risk categories and incident MACE status (n=1,210) in subsamples of the UKB.

### Development of AtheroBurden proteomic signatures

**Extended Figure 4.**
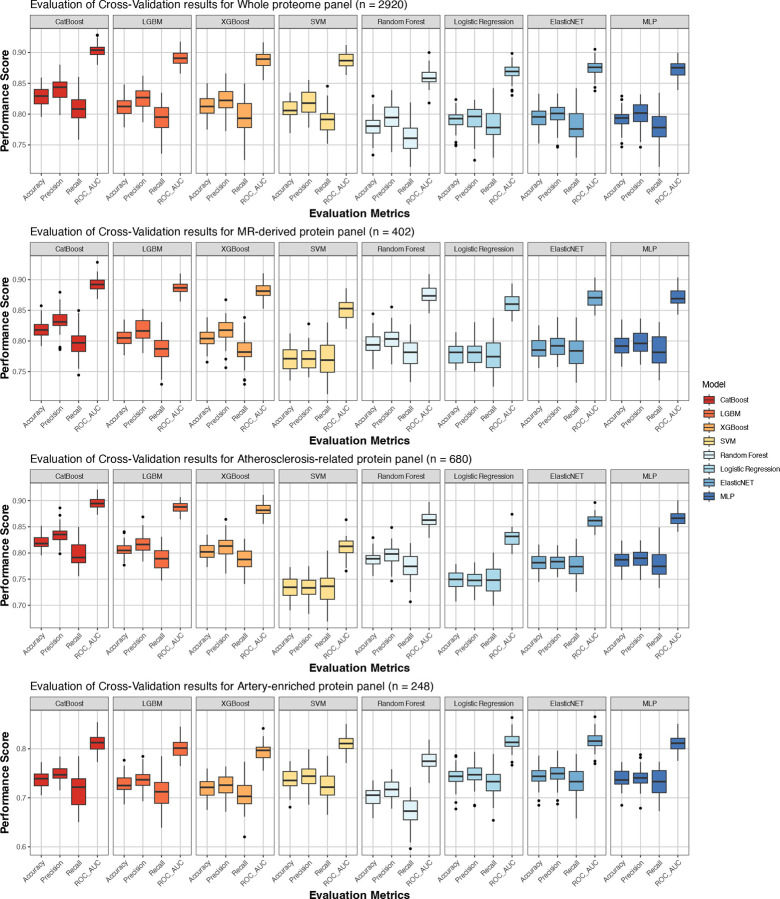
Systematic assessment of machine learning model performance through cross-validation analysis across protein panels. Performance metrics from 10 iterations of 5-fold cross-validation comparing eight machine learning algorithms (CatBoost, LGBM, XGBoost, SVM, Random Forest, Logistic Regression, ElasticNET, and MLP) across four biologically informed protein panels (whole proteome, MR-derived, atherosclerosis-related, and artery-enriched). Box plots depict the distribution of accuracy, precision, recall, and ROC-AUC metrics, where boxes represent the interquartile range (IQR, 25th to 75th percentiles), center lines indicate medians, whiskers extend to 1.5×IQR, and points beyond whiskers denote individual outliers. Abbreviations: ML, machine learning; ROC-AUC, receiver operating characteristic-area under the curve; IQR, interquartile range; ElasticNET, elastic net regression; MLP, multilayer perceptron; SVM, support vector machine; LightGBM, light Gradient Boosting Machine; CatBoost, categorical boosting; XGBoost, eXtreme gradient boosting.

**Extended Figure 5.**
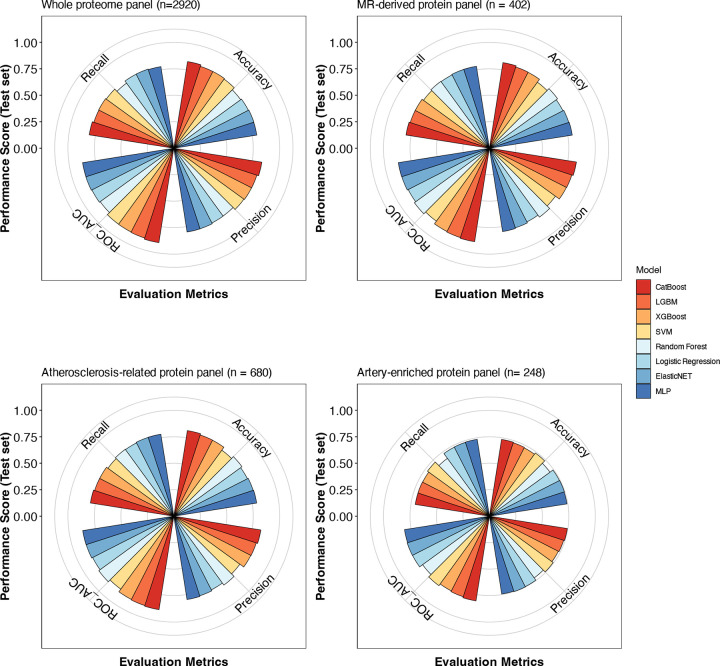
Comparative analysis of machine learning model performance metrics in the testing set across biologically informed protein panels. Radar plots depicting comprehensive performance assessment of eight machine learning algorithms (CatBoost, LGBM, XGBoost, SVM, Random Forest, Logistic Regression, ElasticNET, and MLP) evaluated on independent testing sets across four protein panels. The multi-dimensional visualization integrates five key performance metrics: accuracy, precision, recall, F1 score, and ROC-AUC. Abbreviations: ROC-AUC, receiver operating characteristic-area under the curve; ElasticNET, elastic net regression; MLP, multilayer perceptron; SVM, support vector machine; LightGBM, light Gradient Boosting Machine; CatBoost, categorical boosting; XGBoost, eXtreme gradient boosting.

To construct proteomic signatures of atherosclerosis burden, we developed ML classifiers using a case-control discovery dataset comprising 1,666 participants with an established diagnosis of atherosclerotic cardiovascular disease and 1:1 age- and sex-matched controls (median age 63 years [IQR: 59–66 years], 30% female, **Supplemental Table S1**). Atherosclerotic disease was defined by diagnostic codes encompassing coronary, cerebrovascular (including carotid), aortic, and peripheral arterial manifestations (see Methods). We evaluated the diagnostic performance of eight ML models—Logistic Regression, Random Forest, elastic net regression (ElasticNET), multilayer perceptron (MLP), support vector machine (SVM), light Gradient Boosting Machine (LightGBM), categorical boosting (CatBoost), and eXtreme Gradient Boosting (XGBoost)—using four sets of proteins. The four protein sets were selected to represent different levels of biological relevance to atherosclerosis (Extended Figure 3): (i) the whole proteome (2,920 proteins); (ii) 402 proteins with evidence of causal association with genetic predisposition to coronary artery disease as derived from Mendelian randomization (MR) analyses (MR-derived panel); (iii) 680 proteins coded by atherosclerosis-related genes as curated from literature-based evidence according to the EnrichR platform^42^ (atherosclerosis-related panel); and (iv) 248 proteins overexpressed in the aorta, coronary or tibial arteries, as detected in transcriptomic analyses across 54 tissues in GTEx^43^ (artery-enriched panel). The list of proteins included in every set is provided in **Supplemental Table S2**. Across ten iterations of five-fold cross-validation, CatBoost consistently outperformed the other tested models in accuracy, precision, discrimination, and recall (Extended Figure 4, **Supplemental Table S3**). While MLP and ElasticNET achieved higher performance than CatBoost for the artery-enriched panel in certain iterations, their results were inconsistent and exhibited significant variability. In contrast, CatBoost demonstrated robust and stable performance across all four panels, maintaining superior accuracy and reliability compared to other models (Extended Figure 4, **Supplemental Table S3**). CatBoost also outperformed all other models in accuracy in the testing set (Extended Figure 5, **Supplemental Table S4**). We therefore selected CatBoost-derived models for subsequent analyses.

**Figure 2.**
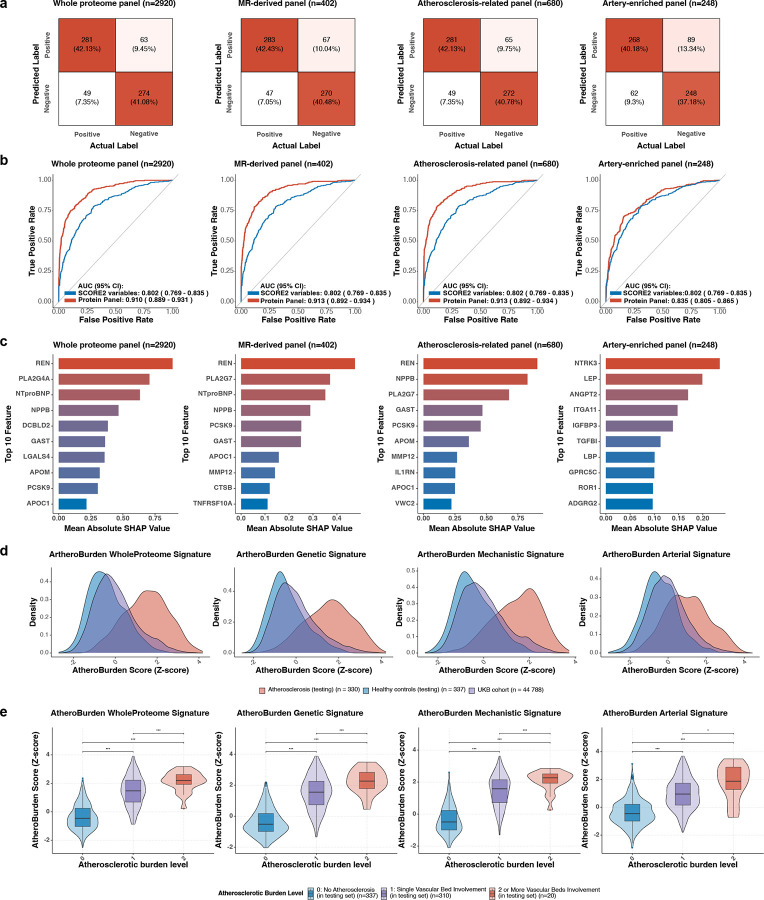
Evaluation of machine learning-derived proteomic signature for atherosclerosis detection and burden assessment across four protein panels. (a) Confusion matrices of classification performance across protein panels. The confusion matrices summarize the classification outcomes for each protein panel, illustrating proportions of true positives, true negatives, false positives, and false negatives. These results reflect the overall accuracy and error distribution of the models. (b) ROC curves for model evaluation. The ROC curves in the testing set illustrate the predictive performance of each protein panel. Each plot includes two curves: one representing the performance of the respective protein panel and the other showing the predictive capacity of cardiovascular risk factors included in the SCORE2 algorithm, used as a comparator. The AUC values and their 95% CI are reported for each curve. (c) Shapley (SHAP) values identify the top 10 contributing proteins. The bar plots display the mean absolute SHAP values for the top 10 proteins contributing to each model, ranked in descending order. (d) Density distributions of AtheroBurden signatures stratified by atherosclerotic status. The density plots depict the distributions of AtheroBurden signatures for healthy controls versus atherosclerotic cases, as well as within the UKB cohort. (e) Violin-box plots of AtheroBurden signatures stratified by the number of affected vascular beds as a measure of atherosclerotic burden (*p < 0.05, ***p < 0.001 between indicated groups). The middle line represents the median, boxes indicate the IQR (25th to 75th percentiles), and whiskers extend to 1.5 times IQR. Abbreviation: MR, Mendelian randomization; ROC, receiver operating characteristic; AUC, area under the receiver operating characteristic curve; CI, confidence interval; SCORE2, Systematic COronary Risk Evaluation version 2; SHAP, SHapley Additive exPlanations; UKB, UK Biobank; IQR, interquartile range.

As shown in Figure 2a, the selected CatBoost models achieved high true positive and true negative rates across all panels in the testing set. Compared to a baseline model using SCORE2 variables (area under the receiver operating characteristic curve [ROC-AUC]: 0.80), the proteomic panels significantly improved discrimination. The atherosclerosis-related, MR-derived, and whole proteome panels achieved comparable enhancements (ROC-AUCs: ~0.91, p < 0.001), while the artery-enriched panel resulted in a modest, non-significant improvement (ROC-AUC: 0.84, p=0.146; Figure 2b). To understand the contributions of individual proteins, we calculated Shapley values (SHAP) across each panel. Renin (REN), NT-proBNP, Natriuretic peptide B (NPPB), and proprotein convertase subtilisin/kexin type 9 (PCSK9) consistently emerged as the top contributors to the atherosclerosis-related, MR-derived, and whole proteome panels (Figure 2c). We subsequently applied these CatBoost models to generate four complementary signatures (AtheroBurden-WholeProteome, -Genetic, -Mechanistic, and -Arterial) for all UKB participants with available proteomic data. The density distributions of all AtheroBurden signatures showed a clear rightward shift in participants with atherosclerotic disease and a corresponding leftward shift in disease-free participants, reflecting higher and lower scores relative to the population mean, respectively (Figure 2d). Furthermore, the signatures captured the burden of atherosclerosis, as illustrated by higher scores among participants with evidence of atherosclerotic disease in two or more versus one arterial bed (Figure 2e).

### Longitudinal associations of AtheroBurden signatures with incident cardiovascular events

**Figure 3.**
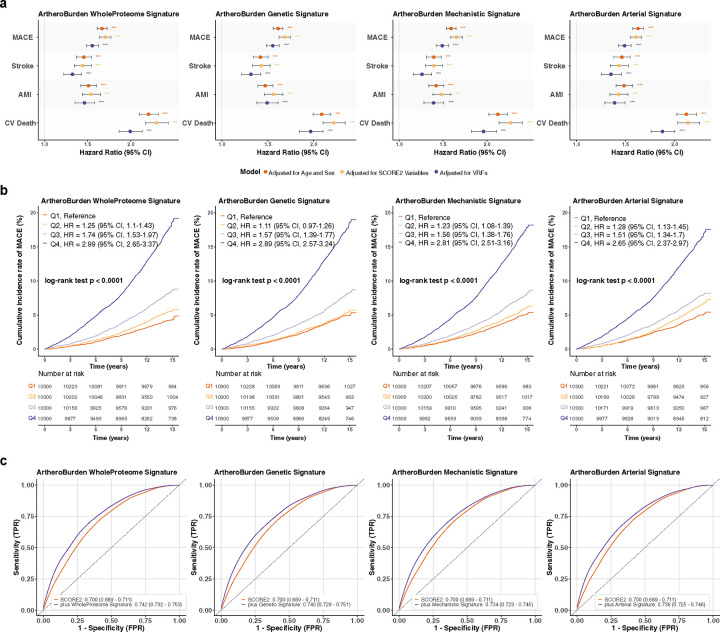
Associations of AtheroBurden scores with future cardiovascular risk in the UK Biobank (n=41,200). (a) Multivariable Cox regression analyses demonstrating associations between AtheroBurden scores and cardiovascular outcomes (MACE and its components—stroke, AMI, and CV death). Effect estimates are presented with 95% confidence intervals under hierarchical adjustment models: demographic factors (age and sex; orange), SCORE2 variables (total cholesterol, HDL-cholesterol, systolic blood pressure, and smoking status; yellow), and VRFs (age, sex, systolic blood pressure, body mass index, smoking status, LDL-cholesterol, triglycerides, estimated glomerular filtration rate, glycated hemoglobin A1c, diabetes, and hypertension status; purple). Statistical significance after FDR adjustment is denoted by asterisks: *p < 0.05, **p< 0.01, ***p < 0.001. (b) Kaplan-Meier curves for the cumulative incidence of MACE stratified by quartiles of AtheroBurden signature. Population risk gradients are illustrated through color-stratified quartiles (Q4: purple; Q1: orange), with hazard ratios adjusted for SCORE2 variables. Risk tables quantify the at-risk population across follow-up intervals. (c) Time-dependent ROC curves evaluating discriminatory performance for predicting cardiovascular risk over a 10-year follow-up period. The orange curve represents the SCORE2 model alone, while the purple curve represents SCORE2 combined with AtheroBurden signatures. The enhancement in risk discrimination is quantified through comparative area under the curve metrics with corresponding 95% confidence intervals. Abbreviations: MACE, major adverse cardiovascular events; AMI, acute myocardial infarction; CV Death, cardiovascular death; HDL, high-density lipoprotein; LDL, low-density lipoprotein; HR, hazard ratio; CI, confidence interval; SCORE2, Systematic COronary Risk Evaluation version 2; VRFs, vascular risk factors; Q1/Q4, quartile 1/quartile 4; ROC, receiver operating characteristic.

To examine the hypothesis that derived signatures capture presence and burden of atherosclerosis among individuals without a history of cardiovascular disease, we assessed associations between the AtheroBurden signatures and incident MACE (composite of acute myocardial infarction [AMI], stroke, or cardiovascular death) were assessed in an independent validation cohort of 41,200 participants (median age 58 years, 56% female, **Supplemental Table S5**). During a median follow-up of 13.7 years, 3,122 incident MACE were documented. All four scores were consistently associated with incident MACE (Figure 3a) in Cox regression models adjusted for age and sex, SCORE2 variables (age, sex, total cholesterol, HDL-cholesterol, systolic blood pressure [SBP], and smoking status), as well as a more comprehensive list of demographic and vascular risk factors (age, sex, SBP, body mass index, smoking status, LDL-cholesterol, triglycerides, estimated glomerular filtration rate, glycated haemoglobin A1c, diabetes, and hypertension status). In the fully-adjusted models, the hazard ratios for MACE per standard deviation increase in the proteomic signatures ranged between 1.49 for the Arterial (95% CI [confidence interval]: 1.43–1.56, p=1.2×10^−70^) and Mechanistic signature (95% CI: 1.42–1.56, p=1.4×10^−58^) to 1.56 for the Genetic (95% CI: 1.49–1.63, p=6.0×10^−81^) and WholeProteome signature (95% CI: 1.48–1.63, p=1.2×10^−77^, **Supplemental Table S6**). All four signatures were significantly associated with all three MACE components, but they showed consistently stronger associations with cardiovascular death than AMI and stroke (Figure 3a and **Supplemental Table S6**). Stratifying the AtheroBurden scores by quartiles, we found strong dose-response relationships, with MACE risk with incidence rates of 17.6–19.2% in the highest (Q4) versus 4.8–5.3% in the lowest quartiles (Q1) at the end of the 16-year follow-up (Figure 3b). The hazard ratio (HR) for Q4 vs. Q1 following adjustments for the full list of vascular risk factors ranged from 2.65 (95% CI: 2.37–2.97) for the Arterial signature to 2.99 (95% CI: 2.65–3.37) for the WholeProteome signature.

**Extended Figure 6.**
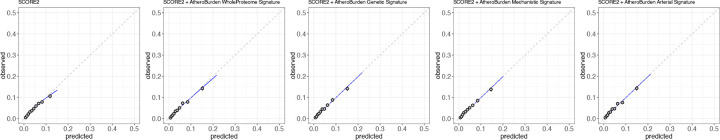
Calibration plots for MACE risk prediction models at 10-year follow-up. This figure presents calibration plots for cardiovascular risk prediction models, illustrating the agreement between predicted and observed risks across four AtheroBurden signatures and SCORE2. Each plot compares the predicted probabilities (x-axis) with the observed probabilities (y-axis) for the SCORE2 model and the SCORE2 model combined with AtheroBurden signatures. The diagonal line represents perfect calibration, indicating complete agreement between predicted and observed risks. The blue lines represent the model’s calibration performance. Abbreviations: MACE, major adverse cardiovascular events; SCORE2, Systematic COronary Risk Evaluation version 2.

**Extended Figure 7.**
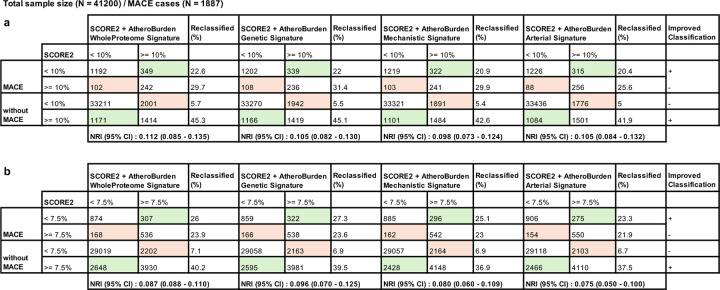
Net reclassification improvement (NRI) for SCORE2 and AtheroBurden signatures in predicting 10-year MACE risk. This table summarizes the NRI results for the combination of SCORE2 and each AtheroBurden signature in predicting 10-year MACE risk. Panel (a) represents the analysis for a predicted risk threshold of 10%, while Panel (b) corresponds to a threshold of 7.5%. The rows show the reclassification percentages for individuals with and without MACE when SCORE2 is combined with each of the four AtheroBurden signatures: whole proteome (AtheroBurden WholeProteome Signature), MR-derived (AtheroBurden Genetic Signature), atherosclerosis-related (AtheroBurden Mechanistic Signature) and artery-enriched (AtheroBurden Arterial Signature). Columns indicate the percentage of individuals reclassified into higher or lower risk categories after the inclusion of AtheroBurden Signatures, along with the total number reclassified. Improvements in classification performance are summarized in the final column, with NRI values and their 95% CI presented below each panel. Green-shaded cells represent reclassifications into more accurate categories, while red-shaded cells indicate potential misclassifications. Abbreviations: NRI, net reclassification improvement; SCORE2, Systematic COronary Risk Evaluation version 2; MACE, major adverse cardiovascular events; MR, Mendelian randomization; CI, confidence interval.

Adding the AtheroBurden signatures to baseline SCORE2 led to significantly improved discrimination for future MACE risk, as indicated by increases in the C-indices (Table 2). The WholeProteome signature exhibited the largest improvement in discrimination, increasing the C-index by 0.04 (from 0.70 to 0.74; p=1.45×10^−68^). These improvements remained robust in sex-stratified analyses, yielding an increase of up to 0.05 in the C-index among males. Testing discrimination changes in 10-year risk, against which SCORE2 is validated, further supported significant improvements (time-dependent ROC-AUC for SCORE2 0.70 vs. 0.74 when adding the AtheroBurden WholeProteome signature, p=1.56×10^−50^, Figure 3c). Incorporating AtheroBurden signatures also led to improvements in calibration, as indicated by improved alignment between predicted and observed risks (Extended Figure 6), as well as in net reclassification improvement (NRI) metrics (category-free net reclassification improvement [cfNRI] and integrated discrimination improvement [IDI]) for both the 10-year and total follow-up periods (p < 0.001 for all comparisons; Table 2). At established clinical decision risk thresholds (7.5% and 10%), addition of AtheroBurden signatures to SCORE2 led to improved reclassification of study participants to the right risk category. For example, the WholeProteome signature improved net reclassification of 11.2% of study participants (95% CI: 8.5%−13.5%) at the 10% risk threshold, while the Genetic signature yielded a 9.6% improvement (95% CI: 7.0%−12.5%) at the 7.5% threshold (Extended Figure 7).

### Association with plaque presence and burden

**Figure 4.**
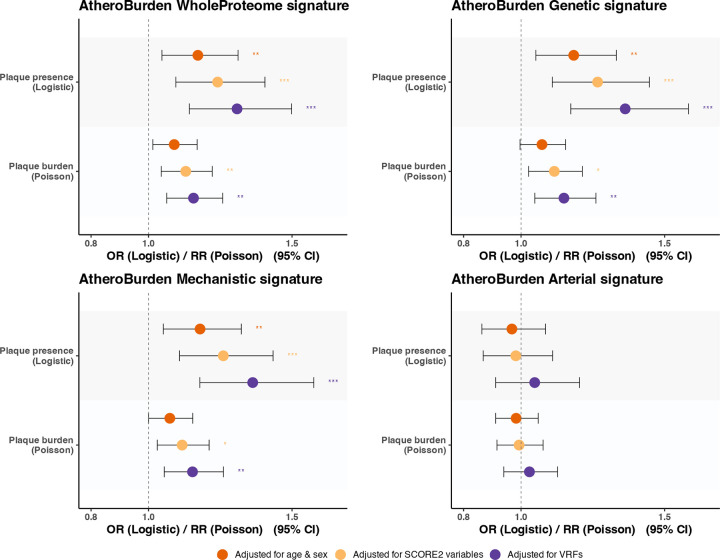
Associations between AtheroBurden scores at baseline and ultrasound-defined carotid plaque presence and burden over follow-up in the UK Biobank (n=1,712). Forest plots illustrating the associations between four AtheroBurden scores and two measures of atherosclerosis: plaque presence (assessed by logistic regression) and plaque count (assessed by Poisson regression). Results are presented as ORs for plaque presence and RRs for plaque burden, each with corresponding 95% CIs. The gray dashed vertical line at 1.0 represents the null hypothesis of no association. Effect estimates are presented with 95% confidence intervals under hierarchical adjustment models: demographic factors (age and sex; orange), SCORE2 variables (total cholesterol, HDL-cholesterol, systolic blood pressure, and smoking status; yellow), and VRFs (age, sex, systolic blood pressure, body mass index, smoking status, LDL-cholesterol, triglycerides, estimated glomerular filtration rate, glycated hemoglobin A1c, diabetes, and hypertension status; purple). Statistical significance after FDR adjustment is denoted by asterisks: *p < 0.05, **p< 0.01, ***p < 0.001. Abbreviations: OR, odds ratio; RR, rate ratio; CI, confidence interval; SCORE2, Systematic COronary Risk Evaluation version 2; VRFs, vascular risk factors; FDR, false discovery rate.

As a next step, we examined associations of the AtheroBurden signatures with imaging evidence of atherosclerosis. As UKB lacks assessment of plaque presence at baseline assessments (2006–2010), we used data from 1,712 individuals who had baseline proteomic measurements and underwent carotid ultrasound imaging at the first follow-up visit starting in 2014. Using a deep learning model that we had previously developed^44^, we found 717 participants to have evidence of carotid atherosclerosis, of whom 222 participants had ≥2 plaques (**Supplemental Table S7**). In logistic regression models for plaque presence and Poisson regression models for plaque count, we found significant associations of the baseline WholeProteome, Genetic, and Mechanistic signatures with carotid plaque presence and burden at the first imaging visit after adjustments for age and sex, SCORE2 variables, and vascular risk factors (Figure 4, **Supplemental Table S8**).

### Longitudinal Assessment of AtheroBurden Signatures and Their Clinical Correlates

To examine whether serial changes in the derived AtheroBurden scores capture progression of atherosclerosis, we conducted a two-step analysis. First, we assessed associations of baseline vascular risk factors with longitudinal score changes in 1,210 UK Biobank participants with at least one follow-up assessment of their circulating proteome at instance 2 (starting 2014) or 3 (starting in 2019). Next, we examined whether individuals who experienced incident MACE showed different progression patterns compared to event-free participants.

**Figure 5.**
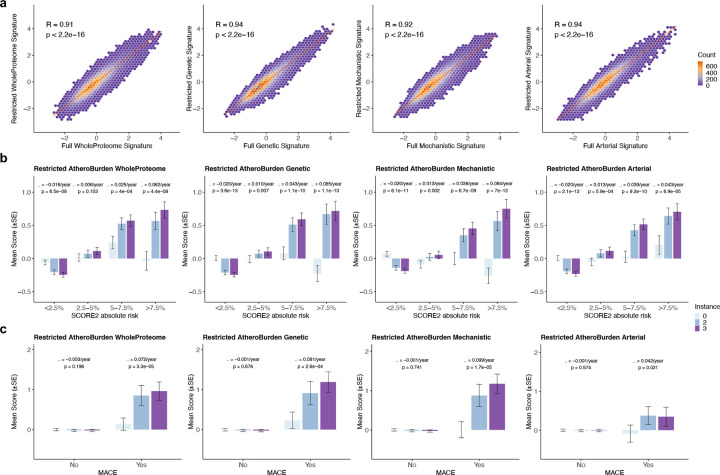
Serial changes of AtheroBurden Scores by baseline cardiovascular risk and incident cardiovascular events in the UK Biobank (n=1,210). (a) Baseline correlation of restricted and full proteomic signatures. Restricted proteomic signatures were derived at baseline (instance 0) using 1,459 proteins common across both available measurement platforms (Olink Explore 1536 and Explore 3072). Scatter plots with hexagonal binning illustrate correlations between restricted and corresponding full AtheroBurden signatures (WholeProteome, Genetic, Mechanistic and Arterial). Pearson correlation coefficients (R) and associated p-values are displayed for each signature. (b) Longitudinal trajectories stratified by baseline SCORE2 risk categories. Temporal evolution of AtheroBurden scores stratified by baseline SCORE2 risk categories (<2.5%, 2.5–5%, 5–7.5%, and >7.5%). Bars represent mean values at three time points (instance 0, 2, and 3), with error bars indicating standard error. Annual progression rates (β) and corresponding p-values were derived from linear mixed-effects models. (c) Temporal evolution of AtheroBurden scores stratified by incident major adverse cardiovascular events (MACE). Mean AtheroBurden scores at three time points (instances 0, 2, and 3) are presented separately for participants without and with subsequent MACE events (denoted as ‘No’ and ‘Yes’, respectively). Error bars represent standard error. Annual progression rates (β) and p-values were derived from linear mixed-effects models. Abbreviations: MACE, major adverse cardiovascular events; SCORE2, Systematic COronary Risk Evaluation version 2; SE, standard error.

Compared to participants with proteomics profiling at a single timepoint, participants with serial assessments were significantly younger (median age at recruitment 49 vs. 58 y) and had a substantially lower burden of vascular risk factors (**Supplemental Table S9**). Because follow-up proteomic assessments at these time points were limited to an earlier version of Olink Explore — covering approximately 50% of the proteins in the newer version— we generated restricted signatures using the overlapping subset of 1,459 proteins (**Supplemental Table S10**). These restricted signatures correlated highly with the full signatures derived at baseline (instance 0), demonstrating robust signal preservation (R=0.91–0.94, all p<2.2×10^−19^; Figure 5a).

When stratified by baseline cardiovascular risk categories, we found individuals in higher baseline cardiovascular risk strata (10-year SCORE2 risk: <2.5%, 2.5–5%, 5–7.5%, and >7.5%) to exhibit steeper annual increases in all four AtheroBurden signatures (Figure 5b). For example, changes in the Genetic AtheroBurden signature ranged from a decrease of 0.020 SD per year (95% CI: −0.026 to −0.015, p = 3.61×10^−13^) in the lowest baseline risk category (<2.5% 10-year risk) to an increase of 0.085 SD per year (95% CI: 0.076 to 0.094; p = 1.15×10^−13^) in the highest risk category (>7.5%). Furthermore, to determine whether AtheroBurden signature progression was specifically associated with clinical outcomes, we tested the progression patterns of individuals who went on experiencing incident MACE during follow-up using linear mixed-effects models, which revealed significantly different trajectories (Figure 5c). Specifically, we found progression of AtheroBurden signatures to be restricted to individuals who experienced MACE during follow-up. Annual progression coefficients in MACE-positive participants ranged from β=0.042 (95% CI: 0.007–0.076; p=0.021) for the Arterial signature to β=0.099 (95% CI: 0.058–0.139; p=1.73×10^−5^) for the Genetic signature, while event-free participants exhibited no significant progression.

### External Validation of AtheroBurden signatures in KORA cohorts

**Extended Figure 8.**
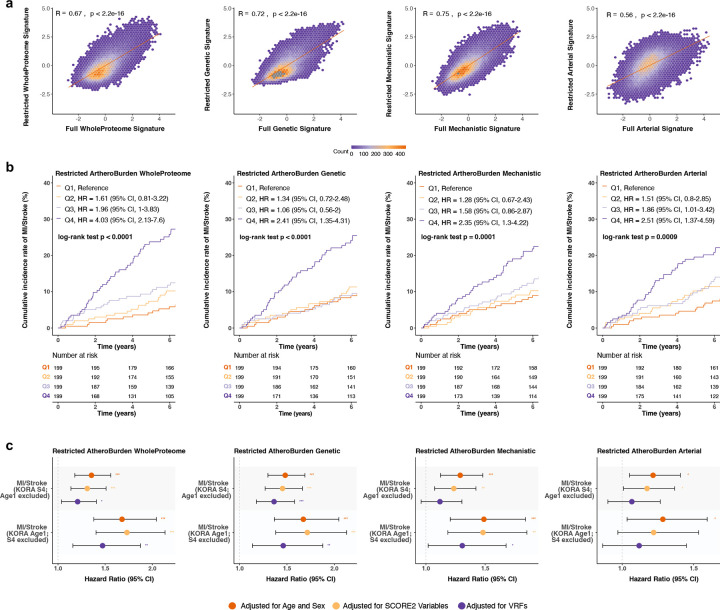
Validation of AtheroBurden Signatures in KORA-Age1 and Non-Overlapping Cohorts. (a) Correlation between restricted (KORA-Age1) and full proteomic signatures in the UK Biobank baseline cohort. Restricted signatures (KORA-Age1) were derived using only proteins quantifiable across all measurement platforms. Hexagonal binning scatter plots demonstrate correlations between restricted and corresponding full signatures with Pearson correlation coefficients (R) and associated p-values. (b) Kaplan-Meier curves showing cumulative incidence of myocardial infarction or stroke stratified by quartiles of restricted AtheroBurden signatures in KORA-Age1. Hazard ratios (adjusted for SCORE2 variables) and log-rank test p-values are displayed with corresponding risk tables. (c) Forest plots show HR and 95% CIs for restricted AtheroBurden signatures in the KORA S4 and KORA-Age1 cohorts after excluding individuals with overlap between the two cohorts. HRs are presented for three adjustment models: demographic factors (age and sex; orange), SCORE2 variables (total cholesterol, HDL-cholesterol, systolic blood pressure, and smoking status; yellow), and VRFs (age, sex, systolic blood pressure, body mass index, smoking status, LDL-cholesterol, triglycerides, estimated glomerular filtration rate, glycated hemoglobin A1c, diabetes, and hypertension status; purple). The gray dashed line represents an HR of 1.0 (no association). Statistical significance is indicated with asterisks: *p < 0.05, **p < 0.01, ***p < 0.001. Abbreviations: HR, hazard ratio; CI, confidence interval; KORA, Cooperative Health Research in the Region of Augsburg; SCORE2, Systematic COronary Risk Evaluation version 2; VRFs, vascular risk factors.

**Figure 6.**
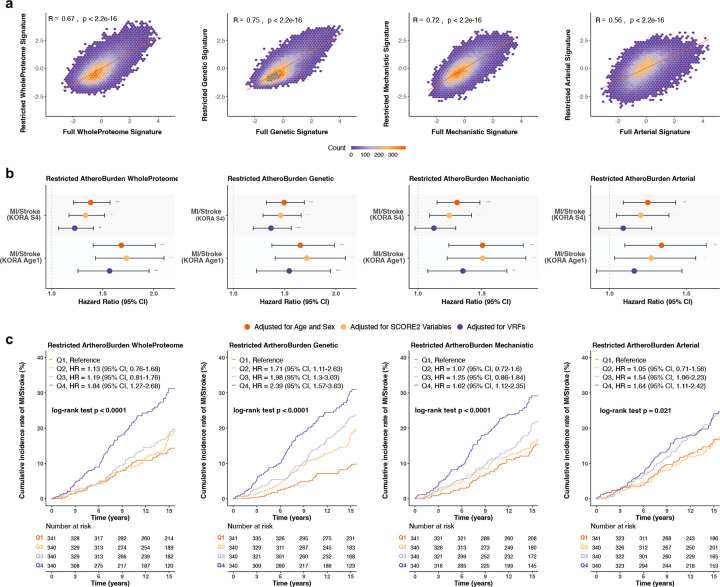
Associations of AtheroBurden signatures with future cardiovascular risk in the KORA S4 (n=1,361) and KORA-Age1 (796) cohorts. (a) Correlation between restricted (KORA S4) and full proteomic signatures in the UK Biobank baseline cohort. Restricted signatures (KORA S4) were derived using only proteins quantifiable across all measurement platforms. Hexagonal binning scatter plots demonstrate correlations between restricted and corresponding full signatures with Pearson correlation coefficients (R) and associated p-values. (b) Forest plots present HRs and 95% CIs for restricted AtheroBurden scores across the KORA S4 and Age1 cohorts. HRs are shown for three adjustment models: demographic factors (age and sex; orange), SCORE2 variables (total cholesterol, HDL-cholesterol, systolic blood pressure, and smoking status; yellow), and VRFs (age, sex, systolic blood pressure, body mass index, smoking status, LDL-cholesterol, triglycerides, estimated glomerular filtration rate, glycated hemoglobin A1c, diabetes, and hypertension status; purple). The grey dashed line indicates an HR of 1.0 (no association). Statistical significance is indicated by asterisks: *p < 0.05, **p < 0.01, ***p < 0.001. (c) Kaplan-Meier curves showing cumulative incidence rates of MI/stroke stratified by quartiles of four restricted AtheroBurden signatures in KORA S4. The HRs displayed are adjusted for SCORE2 variables. Risk tables are provided below each plot, and log-rank test p-values are displayed for group comparisons. Abbreviations: MI, myocardial infarction; HR, hazard ratio; CI, confidence interval; KORA, Cooperative Health Research in the Region of Augsburg; SCORE2, Systematic COronary Risk Evaluation version 2; VRFs, vascular risk factors.

Finally, to externally validate our findings, restricted versions were applied to the population-based KORA S4 (n=1,361) and KORA-Age1 (n=796) prospective cohort studies (**Supplemental Table S11**). Because protein quantification in KORA cohorts was limited to cardiovascular and inflammation panels, restricted signatures were constructed using available overlapping proteins: 232 for WholeProteome, 44 for Genetic, 146 for Mechanistic, and 30 for Arterial signatures in KORA S4; and 242 for WholeProteome, 45 for Genetic, 147 for Mechanistic, and 30 for Arterial signatures in KORA-Age1. There were moderate to strong correlations between the KORA-adapted signatures and the full AtheroBurden signatures in UKB (R=0.56–0.75, all p<2.2×10^−16^) in both cohorts (Figure 6a for S4, Extended Figure 8a for Age1). Compared to the UKB cohort, participants in the KORA S4 and Age1 cohorts were older (S4: median age 63 years; Age1: median age 76 years) and had a more balanced sex distribution (S4: 50% female; Age1: 53% female, Table 1). Over a median follow-up of 15.1 years in S4 and 6.8 years in Age1, 245 and 112 participants were diagnosed with a myocardial infarction [MI] or stroke, respectively.

In age- and sex-adjusted models, we found all four AtheroBurden signatures to be associated with the risk of MI or stroke in both cohorts (Figure 6b and **Supplemental Table S12**), with the Mechanistic and WholeProteome signatures demonstrating significant associations after adjustment for the full set of vascular risk factors. Sensitivity analyses excluding overlapping participants in S4 and Age1 showed similar results, though with limited statistical power to draw definitive conclusions (Extended Figure 8a, **Supplemental Table S13**). Kaplan-Meier analyses demonstrated that participants in the highest signature quartile (Q4) exhibited significantly elevated cumulative incidence of cardiovascular events compared to lower quartiles in KORA S4 (log-rank p < 0.0001 for all signatures, Figure 6c). Similar patterns were observed in KORA-Age1 (Extended Figure 8b). After adjusting for SCORE2 variables, HRs for Q4 relative to Q1 ranged from 1.62 (95% CI: 1.12–2.35) for the Mechanistic signature to 2.39 (95% CI: 1.57–3.63) for the Genetic signature. Similar to the UKB, adding the AtheroBurden signatures on top of baseline SCORE2 led to improvements in discrimination of MI or stroke in both S4 and Age1 (**Supplemental Table S14)**.

## Discussion

In this study, by leveraging large-scale population-based data, we constructed four plasma proteomic signatures that (i) discriminated between presence and absence of clinically diagnosed atherosclerotic disease, (ii) showed a dose-response relationship with the number of vascular beds affected by atherosclerosis, (iii) strongly predicted future risk of cardiovascular events in disease-free individuals, (iv) correlated with imaging-defined carotid plaque burden, and (v) longitudinally changed according to baseline cardiovascular risk and future MACE occurrence. Our findings demonstrate the utility of ML-derived signatures of plasma proteomics for assessing atherosclerosis burden and estimating cardiovascular risk in disease-free individuals.

Our data provide convergent evidence supporting the potential utility of the AtheroBurden signatures as circulating biomarkers of atherosclerosis burden. First, all four proteomic signatures demonstrated strong discriminative performance in identifying individuals with a history of atherosclerotic disease and correlated with disease burden, as reflected by the number of affected vascular beds. Second, among asymptomatic individuals without evidence of atherosclerotic disease, higher values of all four signatures were associated with substantially increased risks of adverse cardiovascular events in UK Biobank and KORA. Individuals who went on to develop cardiovascular events are expected to have a higher burden of atherosclerosis at baseline. These associations persisted after adjustment for traditional vascular risk factors, indicating that the proteomic signatures may capture additional biological information not reflected in standard risk metrics. Third, we found the signatures to be associated with plaque presence and count in carotid ultrasound – an imaging-based surrogate of subclinical atherosclerosis – further reinforcing their relevance to underlying disease biology. Fourth, longitudinal data across three serial time points over a median follow-up of 12.5 years revealed that AtheroBurden scores track with disease progression. Steeper annual increases were observed among individuals with greater baseline vascular risk and among those who subsequently experienced major cardiovascular events, consistent with the trajectory of atherogenesis. These results collectively support the role of proteomic signatures as dynamic, non-invasive indicators of atherosclerotic burden. Nonetheless, prospective validation in independent cohorts with integrated vascular imaging and proteomic profiling will be essential to confirm their utility as biomarkers of subclinical disease and progression.

We developed four distinct proteomic signatures, each comprising proteins with varying relevance to atherosclerosis. Beyond the Arterial, the Mechanistic, Genetic, and WholeProteome signatures demonstrated comparable performance in detecting atherosclerotic disease and predicting future MACE events. Our approach of not relying solely on the whole-proteome panel aimed to reduce the influence of proteins whose circulating levels may reflect secondary effects of tissue ischemia rather than atherosclerosis progression. Investigating the top-ranked proteins in each panel provides insights into the distinct biological signals captured by our signatures. In the artery-enriched panel, highly-ranked proteins were specific to cardiovascular tissues, such as NTRK3 implicated in cardiac remodeling^45^, leptin involved in energy homeostasis^46^ and linked to subclinical atherosclerosis^47^, and ANGPT2 which has shown prognostic relevance in peripheral artery disease^48^ and intracranial stenotic lesions.^49^ Additional high-ranking proteins are linked to extracellular matrix remodeling, growth factor signaling, and inflammation (ITGA11, IGFBP3, TGF3BI, LBP) and have established roles in cardiovascular pathology: ITGA11 in CAD susceptibility and cardiac fibroblast differentiation^50,51^, IGFBP3 in atherosclerotic plaque stability modulation^52^, and TGFBI and LBP in macrophage-mediated inflammatory responses^53–55^. In contrast, the Mechanistic, Genetic, and WholeProteome panels predominantly prioritized systemic cardiovascular markers, including NT-proBNP and REN, as well as cholesterol-associated proteins like PCSK9, and APOC1, potentially reflecting end-organ dysfunction and high cardiovascular risk.

If validated, plasma proteomic signatures of atherosclerosis could have two key translational applications. First, they could enable monitoring of atherosclerosis progression and cardiovascular risk in the context of primary prevention. Unlike imaging techniques, circulating biomarkers are scalable in primary care settings and could be used to screen for advanced atherosclerosis, track cardiovascular risk over time, and monitor responses to preventive interventions. While current proteomics assays are costly, the development of targeted protein panels that capture most of the variance in the full signatures could reduce costs and promote clinical implementation. Second, proteomic signatures may have utility in drug development, both as for patient stratification tools and as surrogate endpoints of efficacy in trials of atheroprotective treatments. Our preliminary analysis in a small subset with serial measurements suggests that these signatures may reflect atherosclerosis progression over time, but whether they respond to treatment effects remains uncertain. In post hoc analyses of two phase 3 trials testing the GLP-1 receptor agonist semaglutide, randomization to treatment vs. placebo led to significant reductions in proteomic signatures associated with MACE risk.^56^ in the absence of scalable non-imaging-based endpoints for atherosclerosis, proteomic signatures may offer a promising surrogate endpoint for early-phase trials. Incorporating proteomic profiles into phase 3 cardiovascular outcomes trials could enable evaluation of whether such signatures correlated with treatment effects on risk reduction at an individual level.

Our study has several limitations. First, the use of clinical diagnoses as proxies for atherosclerotic disease may have biased our signatures toward higher disease burden. As clinical diagnoses typically reflect stenotic atherosclerotic lesions, it remains unknown whether our signatures also capture very early atherosclerotic changes. Although supplementary analyses incorporating carotid plaque phenotyping were reassuring, the ultrasound assessments in UKB were only performed 8 years after the initial proteomic profiling. Future studies that integrate comprehensive vascular imaging with contemporary proteomic profiling could enable more precise phenotyping for model development. Second, external replication in KORA cohorts was limited by reduced protein coverage and differences in cohort characteristics compared to the UKB, potentially affecting direct comparability with our primary findings. Still, restricted signatures based on overlapping proteins showed moderate to strong correlations with the full signatures (R=0.56–0.75) and remained significantly associated with future myocardial infarction and stroke events. While the KORA S4 cohort provided sufficient power for robust Cox regression analyses, replication in the smaller and older KORA-Age1 cohort was limited by reduced sample size and event count. Third, although our study included longitudinal analyses, repeated proteomic measurements in UKB were only available for a small, relatively healthy subset of 1,210 participants from the COVID-19 repeat imaging study. Only 30 MACE events occurred after the second proteomic assessment (instance 2), limiting statistical power to directly assess the relationship between signature trajectories and future cardiovascular risk. Nevertheless, we observed significantly divergent trajectories between participants with and without incident MACE, as well as across SCORE2 risk categories. For these longitudinal assessments, differences in proteomic coverage between time points (1,463 proteins in instances 2/3 vs. 2,923 in instance 0) were addressed by using restricted signatures that preserved the variance of the full signatures (R=0.91–0.94). Fourth, the predominantly European ancestry of the study populations may limit generalizability to other ethnic groups. Future studies in more diverse cohorts are needed to assess the transferability and robustness of the identified signatures across ancestries. Fifth, the selection of proteins for the artery-enriched signature was based on gene expression profiles from the GTEx database^57^, which includes bulk tissue samples from coronary, aortic, and tibial arteries of donors aged 20–71 years who died due to any cause. As these tissues were not selected specifically for atherosclerosis involvement, the resulting protein panel may lack specificity for proteins derived from atherosclerotic plaques. This limitation could partly explain the relatively modest performance of the artery-enriched signature in predicting future MACE and carotid plaque presence compared to the other protein scores. Future studies leveraging proteomic or transcriptomic data directly from human atherosclerotic plaque tissue may yield more atherosclerosis-specific signatures.

In conclusion, plasma proteomic signatures can effectively capture atherosclerosis burden, improving cardiovascular risk prediction in asymptomatic individuals. If replicated in cohorts bridging extensive vascular phenotyping with proteomic profiling, our results suggest that the circulating proteome could serve as an accessible alternative to imaging-based assessments of atherosclerosis. This approach could enable broader implementation of screening and prevention strategies for cardiovascular disease.

## Methods

### Data Sources and Participants

The UK Biobank represents a prospective cohort study encompassing 502,421 individuals from the general UK population.^58^ Between March 2006 and October 2010, participants aged 37–73 years attended one of 22 assessment centres across Scotland, England, and Wales.^58,59^ Each participant completed a touchscreen questionnaire, had physical measurements taken, and provided blood, urine, and saliva samples at baseline. Detailed information about the UKB protocol can be found at http://www.ukbiobank.ac.uk. Participants with available plasma proteomics data were included, excluding those with more than 30% missing values across all measured proteins, resulting in a final study population of 44,788.

1. Discovery dataset (Case–control study)
  The discovery dataset, used to develop the AtheroBurden scoring system, included cases with established atherosclerotic disease (detailed in the “Atherosclerosis Ascertainment“ section) and matched controls without a diagnosis of atherosclerotic disease. To optimally balance the number of available control candidates while maintaining strict age- and sex-matching criteria, participants were initially matched in a 1:6 ratio using a propensity score,^60^ facilitated by the R package *MatchIt (v 4.5.5)*.^61^ From each set of six matched controls, we selected the control with the fewest International Classification of Diseases, 10th Revision (ICD-10) diagnosis codes to minimize comorbidity differences. This approach resulted in a final 1:1 matched pairs, thereby minimizing potential confounding from comorbidity burden while ensuring optimal case-control comparability.
2. Internal validation dataset (Prospective cohort study)
  To assess the performance of the AtheroBurden scores developed from the discovery dataset, we analyzed data from UKB participants with proteomics data and no history of atherosclerotic cardiovascular disease that were not included in the discovery dataset. We included individuals aged 40 to 70 years, resulting in 41,200 participants. Assuming that a score capturing atherosclerosis burden in asymptomatic individuals should be associated with future risk of MACE, we assessed associations of AtheroBurden scores with the occurrence of incident MACE over a median follow-up of 13.7 years.
3. Longitudinal analysis dataset in UKB
  To evaluate temporal changes in AtheroBurden scores and their associations with cardiovascular risk profiles, we utilized repeated proteomic measurements available for a subset of UKB participants. A total of 1,210 individuals, derived from the COVID-19 repeat imaging study, had at least two proteomic measurements, allowing for longitudinal analysis.
4. Carotid plaque subset in UKB
  To assess associations of the developed AtheroBurden scores with subclinical atherosclerosis burden, we used data from a follow-up imaging visit of UK Biobank participants initiated in 2014. A subset of 82,340 participants underwent carotid ultrasound as part of a comprehensive assessment. From this group, 19,499 individuals provided a total of 177,757 carotid ultrasound images, which were subsequently processed for plaque evaluation. Ultrasound imaging was performed using a standardized protocol to capture bilateral carotid arteries.^62^ A total of 1,712 participants had both carotid ultrasound and plasma proteomics data, enabling the investigation of associations between AtheroBurden scores and carotid plaque presence and burden.

#### KORA cohorts

To externally validate our findings, we utilized data from KORA studies^63^, specifically the KORA S4 and KORA-Age1 cohorts.^31,40^ Ethical approval was granted by the local ethics committee, and all participants provided written informed consent. KORA S4 was conducted between 1999 and 2001, enrolling 4,261 participants, of which 1,361 participants aged 55–74 years were included in our analysis due to the availability of proteomics data and follow-up data. KORA-Age1 recruited 1,079 participants aged 65–93 years, with proteomics and follow-up data available for 796 individuals. Both cohorts were used to investigate the relationship between proteomics and cardiovascular outcomes, specifically incident MI and stroke. Among the 1,361 participants in KORA S4, 207 were also part of the KORA-Age1 cohort, though these participants were assessed at different time points. In sensitivity analyses, we excluded the overlapping participants from both cohorts to ensure independence between datasets.

#### Plasma Proteomics in UKB

Blood samples were primarily collected from UKB participants during their baseline visit (instance 0), with additional samples gathered from members of the UKB Pharma Proteome Consortium and individuals in the COVID-19 repeat-imaging study. Plasma proteome characterization was executed utilizing the antibody-based Olink^®^ Proteomics PEA technology.^64^

At baseline (instance 0), proteomic profiling was performed using the Olink^®^ Explore 3072 platform, which encompasses eight distinct panels: Cardiometabolic, Cardiometabolic II, Inflammation, Inflammation II, Neurology, Neurology II, Oncology, and Oncology II. This comprehensive platform enabled quantification of 2,923 unique proteins across 54,219 participants.^65^ Samples were representative of the broader UKB population, with 93% of European ancestry. Protein levels were provided as Normalized Protein eXpression (NPX) values, generated by log-transforming counts normalized to extension controls.^66^ Assessments indicated that protein expression levels were minimally affected by protein batch, study center, and genetic principal components. Detailed protocols for sample handling, processing, and quality control are available online.^65^ For follow-up assessments (instances 2/3), the Olink^®^ Explore 1536 platform was employed, resulting in measurements of 1,463 proteins. Despite differences in panel coverage between baseline and follow-up assessments, the fundamental profiling technology and quality control procedures remained consistent, ensuring methodological comparability across time points.^67^

After excluding 3 proteins (GLIPR1 from the Oncology II panel, PCOLCE from the Cardiometabolic panel, NPM1 from the Neurology panel) that were missing in more than 30% of participants in the final study cohort, the remaining missing values were imputed using a normal distribution method as previously described.^68^ The mean of this imputation distribution was adjusted by subtracting 1.8 standard deviations from the mean of the abundance distribution of all proteins in one sample. The standard deviation of the imputation distribution was set to 0.3 times the standard deviation of the abundance distribution.

#### Plasma Proteomics in KORA

Proteomics data for both KORA S4 and Age1 cohorts were also measured using Olink^®^ Proteomics PEA technology but only covering 276 protein biomarkers (CVD-II, CVD-III, and inflammation panels). The data processing, including quality control and normalization, was performed by the KORA team as previously described.^31,40^ Proteins with more than 25% of values below the limit of detection (LOD) or with missingness were excluded. For proteins present in multiple panels, the version with fewer values below LOD and a lower inter-assay coefficient of variation was retained. After applying these QC criteria, 233 unique proteins passed QC in KORA S4, while 243 proteins passed QC in KORA-Age1. Due to differences in the specific protein panels used between the KORA cohorts and UKB, 232 proteins from KORA S4 and 242 proteins from KORA-Age1 overlapped with those measured in UKB.

### Outcomes definition

#### Atherosclerosis ascertainment

Clinical diagnoses and surgical records were utilized as proxies to identify individuals with presence of atherosclerotic disease. Atherosclerosis was ascertained by identifying events across multiple vascular beds, including coronary, extra- and intracranial, aortic, peripheral, and other arterial sites. Curated disease phenotypes were defined using clinical diagnosis codes from the International Classification of Diseases, 9th and 10th revisions (ICD-9 and ICD-10), as well as surgical procedure codes from the Office of Population Censuses and Surveys, 4th revision (OPCS4). Diagnosis dates were obtained from linked individual participant data. Incident events due to atherosclerotic disease were ascertained from hospital inpatient data summaries (fields 41270, 41271, 41272) as outlined in **Supplemental Table S15**. Prevalent events were defined as those occurring before the participant’s baseline visit when a blood sample was collected. Individuals with corresponding prevalent events for each outcome were considered as cases. Individuals without any experienced atherosclerotic events at baseline and during follow-up were considered as controls and subsequently underwent propensity score matching to construct the discovery dataset. For each individual, atherosclerotic events were evaluated across the five vascular beds described above. The presence of an event in any vascular bed scored 1 point, resulting in atherosclerotic burden levels ranging from 0 (no events) to 2 (events in two or more vascular beds).

#### MACE outcome definitions

The outcome in the internal validation cohort included incident traditional three-point MACE, which comprised AMI, stroke, and cardiovascular death. ICD-9 and ICD-10 codes for each endpoint are listed in **Supplemental Table S16**, ascertained from linked Hospital Episode Statistics (HES) and death registries. For each participant, follow-up began at their baseline visit to the UKB assessment centre, where clinical information and blood samples were collected. The first occurrence of a MACE event was recorded as the primary endpoint for the composite outcome analysis, ensuring each participant contributed only once. For participants without a MACE event, follow-up was censored at the earliest of non-CV death, or the last available hospital inpatient record (31 October 2022 for England, 31 August 2022 for Scotland, and 31 May 2022 for Wales). Mortality data were available until 31 October 2022, and participants without events were censored on these respective dates. When analysing individual components of MACE (AMI, stroke, and CV death) as separate outcomes, we included each participant’s first occurrence of each specific event type.

In the KORA cohorts, the endpoint was the first validated MI or stroke. MIs were ascertained via the Augsburg MI Registry: events before 31 December 2000 followed WHO-MONICA adjudication, and those from 1 January 2001 used ESC/ACC criteria. MIs outside the registry’s area or age limits (> 74 years, > 84 years from 2009) were identified through follow-up questionnaires and confirmed with hospital or physician records; fatal MI cases were identified through death certificates or autopsy reports. Nonfatal strokes (ischaemic or haemorrhagic) were initially identified through self-reports and validated with medical records, while fatal strokes were identified through death certificates or autopsy reports. KORA S4 participants had their baseline examination in 1999–2001, first follow-up examination in 2006–2008 and second follow-up examination in 2013–2014. Furthermore, postal questionnaires were sent out in 2008–2009 and 2016; KORA-Age1 participants had their baseline visit in 2008–2009, first follow-up visit in 2012 and a postal questionnaire was sent to them in 2016.

#### Carotid Plaque Assessment

Carotid ultrasound images were analysed using the deep learning model described by Omarov et al.,^44^ which assesses carotid plaque presence and the number of plaques in the left and right carotid arteries. Plaques were defined as focal protrusions into the arterial lumen with a thickness greater than 50% of the surrounding carotid intima-media thickness,^69^ with plaque presence determined by the identification of at least one plaque in either carotid artery. Plaque burden was assessed based on the total number of plaques detected in both carotid arteries and categorized as 0 for participants with no plaques, 1 for those with a single plaque, and 2 for those with two or more plaques.

### Covariates and SCORE2 Calculation

#### Demographic and Covariates

Baseline variables used in our analyses included age, sex, smoking status (categorized as current, former, or never smoker), SBP, diastolic blood pressure (DBP), cholesterol levels, body mass index (BMI), kidney function (estimated glomerular filtration rate, eGFR), Glycated hemoglobin (HbA1c), and history of diabetes and hypertension. Detailed definitions of these variables, including UKB field IDs, are provided in **Supplemental Table S17**. Smoking status was determined based on baseline questionnaire responses. Blood pressure was measured during the baseline visit, and the average of two readings was used. Cholesterol levels, including total cholesterol (TC), low-density lipoprotein cholesterol (LDL-C), high-density lipoprotein cholesterol (HDL-C), and triglycerides were measured from fasting blood samples. BMI was calculated as weight in kilograms divided by height in meters squared (kg/m^2^). Kidney function was assessed using eGFR calculated from serum creatinine and Cystatin C levels using the CKD-EPI equation (2021).^70^ History of diabetes and hypertension was assessed based on medication use and hospital records. Information on the use of glucose-lowering medications, antihypertensive medications, and lipid-lowering medications was obtained from participant medication data. Hospital records were reviewed to identify prior diagnoses using relevant ICD-9 and ICD-10 codes, with specific codes provided in **Supplemental Table S18**. For the KORA cohorts, similar demographic and clinical variables were collected and defined as previously described.^71,72^

#### SCORE2 Calculation

We estimated the 10-year risk of MACE for each participant using the SCORE2 algorithm,^73^ based on individual factors such as age, sex, SBP, TC, HDL-C, and smoking status. Participants aged 40 to 70 years without MACE were included in this analysis. The linear predictor for each participant was calculated using sex-specific regression coefficients from the SCORE2 working group.^73^ To better align observed and predicted risk, we applied log hazard ratios from the SCORE2 sensitivity analysis that specifically excluded UK Biobank participants (as reported in Supplementary Table 8 of the SCORE2 publication).^73^ For absolute risk calculation, following the approach described in previous studies^74^, these linear predictors were converted into calibrated 10-year risks using the SCORE2 recalibration formula with scaling factors for low-risk European regions (as reported in Supplementary methods Table 4 of the SCORE2 publication).^73^

### ML Model Development

To explore the potential of plasma proteomics to deliver novel biomarker signatures for atherosclerosis, we developed the AtheroBurden scoring system using ML classifiers in the discovery dataset. The process involved selecting relevant protein features, constructing diagnostic models using various ML algorithms, evaluating their performance, and generating continuous AtheroBurden scores from the best model.

#### Protein Feature Pre-selection

To leverage atherosclerosis biology while minimizing confounding from late-stage organ damage signals, we designed four protein panels:

1. Whole Proteome Panel
  Without prior feature selection, we included all 2,920 plasma proteins to construct an ML model predicting the probability of atherosclerosis presence.
2. MR-Derived Protein Panel
  We conducted a two-sample MR analysis to identify proteins that are genetically influenced by predisposition to CAD, providing causal evidence for their potential roles in atherosclerosis pathways, with CAD serving as the exposure and plasma protein levels as the outcome. Genetic instruments were selected from the largest available CAD Genome-Wide Association Study (GWAS) summary statistics by Aragam et al.,^75^ filtered for genome-wide significance (*p* < 5e-08), and further clumped to retain independent variants (r^2^ < 0.001, 10,000 kb window). These instruments were then matched to the Coronary ARtery DIsease Genome-wide Replication and Meta-analysis plus the Coronary Artery Disease Genetics (CARDIoGRAMplusC4D) 1000 Genomes-based GWAS summary statistics.^76^ This dataset does not include UKB data, ensuring that there was no overlap between exposure and outcome datasets. After filtering and matching, 217 SNPs were selected for the MR analysis, with all necessary data, including beta values, obtained from the CARDIoGRAMplusC4D. Plasma protein data were sourced from the UKB Pharma Proteomics Project, which measured 2,940 plasma proteins in 54,219 participants. GWAS summary statistics for these data are publicly available via Synapse.^66^ (https://www.synapse.org/Synapse:syn51365303) The MR analysis was conducted using the R package *TwoSampleMR (v 0.5.6)*, employing the random effect inverse variance weighted (IVW) method for estimating causal effects. Using these data, the MR analysis identified 402 proteins (p <0.05) whose levels were genetically influenced by predisposition to CAD, suggesting their potential role as causal mediators in the disease pathway.
3. Atherosclerosis-Related Protein Panel
  To identify proteins specifically associated with atherosclerosis, we utilized the Enrichr platform,^42^ querying relevant terms and pathway databases for gene sets related to atherosclerosis. This search yielded 52 gene sets, from which we compiled a comprehensive list of genes (n = 3312) associated with atherosclerosis. These annotations were then mapped to the UKB Olink proteome, resulting in 680 atherosclerosis-related proteins, which constituted the atherosclerosis-related protein panel used for model development.
4. Artery-enriched protein panel
  This panel focused on proteins with specific or elevated expression in arterial tissues, hypothesized to be closely related to atherosclerotic lesions. We obtained gene expression data from the Genotype-Tissue Expression (GTEx) project (Release V8, dbGaP Accession phs000424.v8.p2),^57^ which provided comprehensive tissue-specific bulk RNA seq expression profiles across various human tissues, including vascular tissues. We grouped three vascular tissues—coronary, aorta, and tibial artery—together as the vascular group, while all other organs were grouped as the non-vascular group. Genes were considered artery-enriched if their expression levels in the vascular group were at least threefold higher than in the non-vascular group. We then mapped these genes to the plasma proteomics data from the UKB, resulting in an artery-enriched protein panel of 248 proteins used for model development.

#### ML Classifiers

We utilized Python packages *scikit-learn (v 1.3.2)*, *catboost (v 1.2.5)*, *lightgbm (v 4.2.0), and xgboost (v 2.0.3)*, to implement a range of ML techniques, including Logistic Regression, Random Forest, ElasticNET, SVM, MLP. Additionally, gradient boosting classifiers such as LightGBM, CatBoost, and XGBoost were employed. These classifiers were designed to predict whether participants belonged to class 1 (diagnosed with atherosclerotic events at baseline) or class 0 (event-free). ML models were established using a discovery dataset created using propensity score matching based on age and sex to select healthy controls (n=1,666) for participants with prevalent atherosclerotic events (n=1,666). This matching technique helps mitigate potential nonlinear confounding effects. We then randomly split the discovery dataset into training (80%) and testing sets (20%), with stratification ensuring balanced distribution of atherosclerotic events in both sets.

All models were trained and validated using ten iterations of five-fold stratified cross-validation on the training set, with the dataset resampled for each iteration to ensure robustness. Performance was evaluated using accuracy, precision, recall, and ROC-AUC, providing comprehensive insights into classification effectiveness and error patterns. Hyperparameters for the cross-validated models were optimized using Optuna,^77^ an automated framework, with each configuration undergoing the same cross-validation strategy. Algorithm-specific search spaces were defined, encompassing learning rates (10^−5^ to 10^−1^), regularization parameters (C values from 10^−5^ to 10), tree depths (3 to 10), number of estimators (50 to 200), and other model-specific parameters. Performance was assessed using average ROC-AUC and other relevant metrics, and optimal hyperparameters were selected based on configurations achieving the highest cross-validated scores. CatBoost was selected as the best-performing model based on its highest average ROC-AUC and stability (consistency of performance across testing sets). Hyperparameter specifications for all evaluated models are provided in **Supplemental Table S19**. To compare the performance of the selected CatBoost model with a traditional risk prediction approach, a separate logistic regression model was developed using SCORE2 variables (age, sex, total cholesterol, HDL-cholesterol, systolic blood pressure, and smoking status) in the discovery dataset, with performance assessed using ROC-AUC. Statistical significance of ROC-AUC differences was evaluated using the DeLong test. Feature importance was assessed using SHapley Additive exPlanations (SHAP) values, which quantify each feature’s contribution to the model’s predictions.^78^

#### Generating continuous AtheroBurden signature

The CatBoost classifier^79^ was constructed using protein expression profiles as input features, with models trained to discriminate between individuals with and without atherosclerotic disease. Following comprehensive hyperparameter optimization through five-fold cross-validation, the final classifier was applied to the entire UKB cohort to generate continuous risk predictions. The raw prediction values obtained directly from the CatBoost algorithm—representing the untransformed linear combination of weighted protein features—were subsequently standardized as Z-scores (centered at zero with a standard deviation of one) to facilitate inter-individual comparability. Four AtheroBurden signatures were derived from the respective protein panels: AtheroBurden-Arterial, based on the artery-enriched panel; AtheroBurden-Mechanistic, based on the atherosclerosis-related panel; AtheroBurden-Genetic, based on the MR-derived panel; and AtheroBurden-WholeProteome, based on the whole proteome panel. All models were trained in the discovery dataset and subsequently applied to the entire UKB cohort.

For longitudinal validation using follow-up measurements and external validation in independent cohorts, restricted versions of the AtheroBurden signatures were generated to address differential protein coverage. For UKB participants assessed at follow-up timepoints (instances 2/3) where the Olink^®^ Explore 1536 platform was employed, restricted signatures were computed by applying the original prediction algorithm with missing value assignments (NA) for proteins not measured on the restricted platform. An identical methodological approach was implemented for external validation in the KORA cohorts. To quantify potential information loss resulting from reduced protein coverage, equivalent restricted signatures were generated in the baseline UKB cohort (instance 0) using only proteins available across all platforms. Correlation analyses were subsequently conducted to evaluate the proportion of variance in the full signatures that could be explained by these restricted protein models.

### Statistical Analysis

Population characteristics were summarized as mean ± SD for normally distributed variables, median (IQR) for skewed variables, and n (%) for categorical variables. Missing clinical data were imputed using predictive mean matching via the R package *mice (v 3.16.0)*. Continuous variables were imputed using predictive mean matching, binary variables via logistic regression, and ordinal variables with a proportional odds model. Imputation was based on age and sex (no missing values) to enhance data quality and repeated five times for robustness. Cox proportional hazards regression models were applied to evaluate the association between AtheroBurden scores and time to MACE among participants without baseline MACE. Three models were constructed: Model 1 adjusted for age and sex. Model 2 further adjusted for TC, HDL-C, SBP, and smoking (based on SCORE2 variables); and Model3 adjusted VRFs included age, sex, SBP, BMI, smoking status, LDL-C, triglycerides, eGFR, HbA1c, diabetes, and hypertension status. Multiple comparisons were addressed using FDR correction to control for type I error.

To assess the added value of AtheroBurden signatures over SCORE2, we evaluated discrimination improvement using concordance indices (C-index), calculated with the concordance.index function (*survcomp package, v 1.52.0*, R). C-index differences (ΔC-index) were compared using the cindex.comp function, reporting p-values and 95% CI. Time-dependent ROC curves were generated at 10-year follow-up points to track model performance over time. Kaplan-Meier survival curves were used to estimate cumulative MACE incidence across AtheroBurden signature quartiles, with log-rank tests performed for group comparisons. Calibration of the SCORE2 model, with and without AtheroBurden signatures, was evaluated using calibration plots comparing observed 10-year Kaplan-Meier estimates and predicted probabilities within deciles of predicted risk. Reclassification metrics included NRI, cfNRI, and IDI. The NRI analysis employed two established clinical thresholds (7.5% and 10%) derived from SCORE2 risk stratification guidelines, selected based on their validated clinical utility in cardiovascular risk assessment. These thresholds were applied as population-level cut-points to evaluate the overall reclassification performance of proteomic models when added to traditional risk factors. NRI was calculated using the package *nricens (v.1.6)*^80^ with confidence intervals and p-values based on 1000-fold bootstrap standard errors. The cfNRI and IDI metrics were calculated using the *survIDINRI (v.1.1–2)*^81^. Sensitivity analyses examined AtheroBurden signatures’ associations with individual MACE components (AMI, stroke, and CV death) with FDR correction. Logistic and Poisson regressions were employed to assess AtheroBurden signatures’ relationships with carotid plaque presence and burden.

The longitudinal progression of AtheroBurden signatures was assessed using linear mixed-effects models with time since baseline (in years) as a continuous variable. The scores were derived from AtheroBurden scoring systems based on available proteomic data and were repeatedly measured for the same individuals at three time points: baseline (instance 0) and two follow-ups (instances 2 and 3). The mixed-effects models included random intercepts to account for individual-level variability, with fixed effects for standardized baseline risk factors and time.

In the external validation analysis, cox proportional hazards models, adjusted using the same variables as previous analysis, were applied to examine the associations between restricted AtheroBurden signatures and incident MI and stroke. Kaplan-Meier survival curves were applied to estimate cumulative MI and stroke incidence across AtheroBurden scores quartiles in KORA S4, while improvements in discrimination were evaluated by changes in C-index when incorporating restricted AtheroBurden scores into SCORE2 variables across both cohorts. Sensitivity analysis excluded overlapping participants between KORA S4 and Age1 to maintain independence. Statistical power calculations for external validation analyses were conducted using R package *powerSurvEpi* (*v 0.1.3*)^82^, with an alpha level of 0.05.

All statistical analyses were performed using R (version 4.3.3), and ML procedures were conducted in Python (version 3.9.10). A two-sided p <0.05 was considered statistically significant.

## Extended Data

## Supplementary Files

This is a list of supplementary files associated with this preprint. Click to download.

- supTable.xlsx

## Figures and Tables

**Table 1.** Baseline characteristics of participants for each the three cohorts analyzed for this study.

Characteristic	UK Biobank	KORA S4	KORA-Age1
	N = 44,788	N = 1,361	N = 796
Age at recruitment (years), Median (Q1, Q3)	58.00 (50.00, 64.00)	63.00 (59.00, 68.00)	76.00 (70.00, 81.00)
Sex, n (%)			
*Female*	24,234 (54%)	685 (50%)	423 (53%)
*Male*	20,554 (46%)	676 (50%)	373 (47%)
BMI (kg/m^2), Median (Q1, Q3)	26.78 (24.19, 29.90)	27.95 (25.65, 30.83)	27.93 (25.51, 30.70)
Diastolic blood pressure (mmHg), Median (Q1, Q3)	82.00 (75.00, 89.00)	80.00 (73.50, 87.50)	76.00 (69.50, 83.00)
Systolic blood pressure (mmHg), Median (Q1, Q3)	138.00 (126.00, 152.00)	135.00 (121.50, 148.00)	138.00 (124.50, 150.50)
Cholesterol (mmol/L), Median (Q1, Q3)	5.62 (4.89, 6.38)	6.27 (5.53, 6.97)	5.53 (4.81, 6.15)
HDL Cholesterol (mmol/L), Median (Q1, Q3)	1.40 (1.18, 1.67)	1.44 (1.19, 1.75)	1.45 (1.20, 1.68)
LDL Cholesterol (mmol/L), Median (Q1, Q3)	3.50 (2.94, 4.08)	3.94 (3.27, 4.60)	3.28 (2.76, 3.90)
Triglycerides (mmol/L), Median (Q1, Q3)	1.49 (1.06, 2.14)	1.36 (0.99, 1.93)	1.41 (1.02, 1.99)
eGFR (ml/min/1.73m^2), Median (Q1, Q3)	95.06 (84.89, 104.57)	84.50 (74.73, 92.29)	71.70 (59.43, 83.03)
HbA1c (mmol/L), Median (Q1, Q3)	35.30 (33.00, 38.00)	37.71 (35.52, 40.98)	37.71 (35.52, 40.98)
Previous smoking, n (%)	15,806 (35%)	510 (38%)	307 (39%)
Current smoking, n (%)	4,816 (11%)	187 (14%)	34 (4%)
Blood Pressure Medication, n (%)	10,384 (23%)	455 (34%)	535 (67%)
Cholesterol Lowering Medication, n (%)	8,152 (18%)	134 (9.9%)	199 (25%)
Diabetes, n (%)	2,009 (4.5%)	89 (6.6%)	112 (14%)
Hypertension, n (%)	11,068 (25%)	737 (54%)	599 (75%)

Abbreviations: KORA, Cooperative Health Research in the Region of Augsburg; BMI, body mass index; LDL, low density lipoprotein; HDL, high density lipoprotein; HbA1c, glycated hemoglobin A1c; eGFR: estimated glomerular filtration rate.

**Table 2:** Incremental discrimination and reclassification improvement for predicting MACE with the addition of AtheroBurden signatures. Comparison of model discrimination (C-index, ΔC-index) and reclassification (cfNRI, IDI) when adding various AtheroBurden signatures to SCORE2 for predicting major adverse cardiovascular events (MACE). Results include internal validation and subgroup analyses by sex. All comparisons use SCORE2 as reference (Ref).

Internal validation dataset (N=41,200; 3,122 incident MACE cases)				10-year follow up (1,887 incident MACE cases)	
model	C-index (95% CI)	ΔC-index (vs. SCORE2)	p value	cfNRI (95% CI)	IDI (95% CI)
SCORE2	0.701 (0.683–0.718)	Ref	-	Ref	Ref
SCORE2 + AtheroBurden WholeProteome	0.737 (0.729–0.845)	0.036	1.45E-68	0.172 (0.147–0.194)	0.018 (0.014–0.021)
SCORE2 + AtheroBurden Genetic	0.735 (0.727–0.743)	0.034	1.32E-65	0.177 (0.153–0.201)	0.017 (0.015–0.021)
SCORE2 + AtheroBurden Mechanistic	0.730 (0.722–0.739)	0.030	5.96E-54	0.164 (0.137–0.187)	0.014 (0.011–0.017)
SCORE2 + AtheroBurden Arterial	0.730 (0.722–0.739)	0.030	1.18E-48	0.155 (0.132–0.183)	0.018 (0.014–0.021)
Analysis in females (N=18,057 1,192 incident MACE cases)				10-year follow up (681 incident MACE cases)	
SCORE2	0.722 (0.709–0.736)	Ref	_-_	Ref	Ref
SCORE2 + AtheroBurden WholeProteome	0.746 (0.733–0.759)	0.023	7.00E-20	0.124 (0.086–0.169)	0.008 (0.005–0.012)
SCORE2 + AtheroBurden Genetic	0.745 (0.732–0.758)	0.023	1.00E-19	0.137 (0.100–0.172)	0.008 (0.004–0.012)
SCORE2 + AtheroBurden Mechanistic	0.743 (0.729–0.755)	0.020	9.00E-17	0.122 (0.078–0.161)	0.006 (0.003–0.010)
SCORE2 + AtheroBurden Arterial	0.743 (0.729–0.756)	0.020	3.00E-14	0.121 (0.088–0.162)	0.009 (0.006–0.014)
Analysis in males (N=23,143 1,930 incident MACE cases)				10-year follow up (1,206 incident MACE cases)	
SCORE2	0.681 (0.669–0.692)	Ref	-	Ref	Ref
SCORE2 + AtheroBurden WholeProteome	0.729 (0.718–0.740)	0.049	7.00E-48	0.224 (0.198–0.256)	0.031 (0.024–0.039)
SCORE2 + AtheroBurden Genetic	0.730 (0.719–0.741)	0.049	7.00E-51	0.230 (0.203–0.267)	0.032 (0.024–0.040)
SCORE2 + AtheroBurden Mechanistic	0.725 (0.714–0.736)	0.044	2.00E-42	0.227 (0.199–0.252)	0.027 (0.020–0.035)
SCORE2 + AtheroBurden Arterial	0.722 (0.711–0.733)	0.041	1.00E-36	0.196 (0.169–0.229)	0.030 (0.023–0.039)

Abbreviations: cfNRI, category-free net reclassification improvement; NRI, net reclassification improvement; IDI, integrated discrimination improvement; SCORE2, Systematic COronary Risk Evaluation version 2; CI, confidence intervals; Ref, reference; MACE, major adverse cardiovascular events.

## Data Availability

The coronary artery disease GWAS summary statistics are available through the GWAS Catalog (https://www.ebi.ac.uk/gwas/) (accession no. GCST90132314) and CARDIoGRAMplusC4D (https://www.cardiogramplusc4d.org/data-downloads/). The proteomic GWAS summary data from the UK Biobank Pharma Proteomics Project (UKB-PPP) can be accessed publicly via Synapse (https://www.synapse.org/Synapse:syn51365301). To protect patient confidentiality and ensure compliance with consent agreements, individual-level data and proteomic profiles from UK Biobank and KORA are available under controlled access. UK Biobank data can be accessed by approved researchers through the UK Biobank data access framework (https://www.ukbiobank.ac.uk/enable-your-research/apply-for-access), with the full dataset available on the Research Analysis Platform (https://www.ukbiobank.ac.uk/enable-your-research/research-analysis-platform). KORA datasets are available on reasonable request through a project agreement from KORA (https://helmholtz-muenchen.managed-otrs.com/external/). Requests should be sent to kora.passt@helmholtz-munich.de and are subject to approval by the KORA board.
